# Robustness of the performance of the optimized hierarchical two-parameter logistic IRT model for small-sample item calibration

**DOI:** 10.3758/s13428-022-02000-5

**Published:** 2022-11-04

**Authors:** Christoph König, Christian Spoden, Andreas Frey

**Affiliations:** 1 Goethe University Frankfurt, Frankfurt am Main, Germany; 2 University of Applied Sciences Emden/Leer, Emden, Germany

**Keywords:** Bayesian psychometrics, Bayesian hierarchical modeling, Item calibration, Item response theory, Small-sample calibration

## Abstract

Hierarchical Bayesian modeling is beneficial when complex models with many parameters of the same type, such as item response theory (IRT) models, are to be estimated with sparse data. Recently, Koenig et al. (*Applied Psychological Measurement, 44*, 311–326, 2020) illustrated in an optimized hierarchical Bayesian two-parameter logistic model (OH2PL) how to avoid bias due to unintended shrinkage or degeneracies of the posterior, and how to benefit from this approach in small samples. The generalizability of their findings, however, is limited because they investigated only a single specification of the hyperprior structure. Consequently, in a comprehensive simulation study, we investigated the robustness of the performance of the novel OH2PL in several specifications of their hyperpriors under a broad range of data conditions. We show that the novel OH2PL in the half-Cauchy or Exponential configuration yields unbiased (in terms of bias) model parameter estimates in small samples of *N* = 50. Moreover, it outperforms (especially in terms of the *RMSE* of the item discrimination parameters) marginal maximum likelihood (MML) estimation and its nonhierarchical counterpart. This further corroborates the possibility that hierarchical Bayesian IRT models behave differently than general hierarchical Bayesian models. We discuss these results regarding the applicability of complex IRT models in small-scale situations typical in psychological research, and illustrate the extended applicability of the 2PL IRT model with an empirical example.

In hierarchical Bayesian models, the specification of the prior distributions for individual parameters of the same type is inferred from the data by hyperprior distributions for their grand means and variances. This hierarchical Bayesian modelling approach is, in theory, beneficial when complex models with many parameters of the same type are to be estimated with sparse data (Betancourt & Girolami, 2015). It makes it possible to use information from all parameters of the same kind to estimate individual parameters, thus maximizing the information contained in a given data set. Therefore, precision in individual parameter estimates is increased, which is typically reflected by narrower 95% highest density intervals (HDIs) compared to parameter estimates obtained with nonhierarchical approaches. The narrower HDIs are the result of a partial pooling process inherent to the hierarchical structure of the prior distributions. As the variance in the individual parameters decreases, their estimates are drawn towards their grand mean, that is, to the mean of their hyperprior distribution (Jackman, 2009).

This is also referred to as shrinkage. Because the variance of the model parameter estimates is seldom zero and is inferred from empirical data (Fox, 2010), individual parameter estimates in hierarchical Bayesian models exhibit a certain amount of bias compared to estimates obtained in non-hierarchical approaches. Since the partial pooling process adapts the amount of shrinkage to the variance present in the data, the bias in the model parameters should be negligible as long as the variance components of the parameters are estimated accurately. If the variance components either are under- or overestimated, the amount of shrinkage will be incorrect and lead to biased model parameters (especially in case of underestimated variance components). Thus, the hyperprior distributions for the parameter variances play a pivotal role in the performance of hierarchical Bayesian models. Another potential source of bias in hierarchical Bayesian models is caused by the dependencies of the individual parameters with their grand means. These dependencies cause *funnel degeneracies*, where high-density/low-volume regions are below low-density/high-volume regions (Betancourt & Girolami, 2015). These regions exhibit considerable changes in the curvature of the posterior distribution. This makes it difficult for the Markov chain Monte Carlo (MCMC) sampler, which is used to estimate the model parameters, to explore the posterior distribution efficiently (Betancourt, 2017).

Thus, in order to benefit from the hierarchical Bayesian approach, the hyperprior distributions for the parameter variances must be considered carefully. Although in the methodological literature researchers are frequently discouraged from using the inverse Gamma and inverse Wishart distributions (Alvarez et al., 2016; Gelman, 2006; Polson & Scott, 2012; Simpson et al., 2014), those two distributions are still widely used in Bayesian hierarchical models (e.g., Lu et al., 2020; Tijmstra et al., 2018). The alternative half-Cauchy and Exponential distributions as priors for variance components have been investigated only recently (Koenig et al., 2020; Liu & Yang, 2018; Sheng, 2017). Another aspect that requires careful consideration is the parameterization and complexity of the model at hand. This also applies to item response theory (IRT) models, which are mostly nonlinear and feature many parameters of the same kind, making them ideal candidates for hierarchical Bayesian modeling. Due to their complexity, however, they require rather large sample sizes to obtain accurate person and item parameter estimates, especially when no prior information is available: The two-parameter logistic (2PL) model, for instance, has a recommended sample size of *N* ≥ 500 (De Ayala, 2009; smaller sample sizes are possible when prior information is available). Hence, the applicability of this frequently used class of models to small-scale situations, as they often occur in psychological research, is limited.

To extend the applicability of the 2PL model to small-scale situations, Koenig et al. (2020) illustrated a novel, optimized hierarchical two-parameter logistic (OH2PL) IRT model. More recently, Gilholm et al. (2021) successfully applied the OH2PL to a six-dimensional case with *N* = 115 students. In the OH2PL, Koenig et al. (2020) combined several adjustments with the aim of eliminating bias caused by the choice of the hyperprior distributions and of avoiding the degeneracies of the standard hierarchical 2PL. They showed that it is applicable to situations with 100 respondents. Moreover, the OH2PL outperformed its nonhierarchical counterpart in terms of bias of the item parameter estimates, especially regarding the item discrimination parameters. This is a remarkable finding because it contradicts the theoretical behavior of hierarchical Bayesian models in general. Both results, however, were found only for a single weakly informative specification of the variance-related hyperprior distributions under a small number of carefully selected simulation conditions.

Whether the findings by Koenig et al. (2020) can be generalized to a broader range of data conditions, and beyond a single weakly informative specification of the variance-related hyperprior distributions, is still unclear. The specific focus on the variance-related hyperprior distributions arises from the theoretical proposition that, for complex nonlinear hierarchical models such as IRT models, variance components play a crucial role in the accuracy of item parameter estimates: given partial pooling, the bias in the estimates of variance components relates directly to the bias in the associated item parameter estimates. Moreover, it remains unclear how the non-centered parameterization of the OH2PL responds to different specifications of its variance-related hyperprior distributions.

Consequently, the primary purpose of this study is to investigate the performance of the OH2PL in terms of parameter estimation accuracy in calibration sample sizes below 500 respondents across different specifications of the hyperprior structure of the OH2PL and a broader range of data conditions. More specifically, we aim to answer the following research question: How sensitive is the bias in the parameter estimates of the OH2PL against different specifications of the half-Cauchy, Exponential, and inverse Gamma hyperprior distributions in different sample sizes and test lengths, as well as different variances and correlations of the item parameters? We consider the performance of the OH2PL to be robust if (a) the model parameter estimates are unbiased and (b) if the conditional bias and *RMSE* do not distinctively differ across the specifications of the respective hyperprior distribution. Lastly, we illustrate the advantage of the OH2PL with regard to an increased applicability of the 2PL IRT model to suboptimal testing conditions with an empirical example.

The answer to this research question adds to the literature in three ways. First, it provides guidance on how to specify the half-Cauchy, Exponential, and inverse Gamma distribution properly as weakly informative hyperpriors for variance components in hierarchical Bayesian IRT models. Second, it provides insights into the relationship between the accuracy of the estimated variance components and the respective item parameter estimates in hierarchical Bayesian IRT models. Third, it provides further evidence for the applicability of the OH2PL in very small calibration sample sizes and for the utility of the hierarchical Bayesian approach to IRT modeling in general.

## The optimized hierarchical two-parameter logistic IRT model

Let *y*_*ij*_ ∈ {0, 1} be the response of person *j* to item *i*, and θ_*j*_ the ability of person *j* (the person parameter), which is typically assumed to follow a standard normal prior distribution. Moreover, α_*i*_ is the discrimination of item *i*, and β_*i*_ is its difficulty. Let the logit of a function *x* be defined by

1 $$\textrm{logit}=\frac{\exp (x)}{1+\exp (x)},$$

then the OH2PL is specified by

2 $$\Pr \left({\textrm{y}}_{ij}=1|{\uptheta}_j,{\upalpha}_i,{\upbeta}_i\right)=\textrm{Bernoulli}\left(\textrm{logit}\left[{\upalpha}_i\ \left({\uptheta}_j-{\upbeta}_i\right)\right]\right)$$

3 $${\uptheta}_j\sim \textrm{N}\left(0,1\right)$$

4 $$\overset{\sim }{{\boldsymbol{\upxi}}_i}\sim \textrm{N}\left(0,1\right)$$

5 $${\upmu}_{\upalpha}\sim \textrm{N}\left(0,1\right)$$

6 $${\upmu}_{\upbeta}\sim \textrm{N}\left(0,2\right)$$

7 $${\textbf{L}}_{\boldsymbol{\Omega}}\sim \textrm{LKJ}(2)$$

8 $${\uptau}_{\upalpha_{unif},{\upbeta}_{unif}}\sim \textrm{U}\left(0,\uppi /2\right).$$

The novel OH2PL integrates three different adjustments to the common hierarchical specification of the 2PL model. First, it uses a separation strategy regarding the hyperprior distributions for the correlation between the item parameters and their variance components (Barnard et al., 2000; Ulitzsch et al., 2020). It is based on the Cholesky factor of the correlation matrix of the item parameters **ξ**_*i*_ = {log α_*i*_, β_*i*_}, *i* = 1, …, *I* items, and their variance components τ_α_ and τ_β_. This strategy introduces more flexibility for the specification of these hyperprior distributions. Moreover, it eliminates the *a priori* dependencies between the variance components and the covariances commonly associated with the inverse Wishart specification as the standard distribution for the covariance matrix of the item parameters **Σ**_**ξ**_ (e.g., Alvarez et al., 2016).

Second, the OH2PL uses either the half-Cauchy or the Exponential as the hyperprior distribution for the variance components τ_α_ and τ_β_ of the item parameters, instead of the more commonly applied inverse Gamma distribution. The inverse Gamma distribution has a low mass near zero. It is therefore quite informative even when specified as noninformative and it behaves erratically when the true variance is close to zero (Gelman, 2006). Using either the half-Cauchy or the Exponential distribution eliminates the bias resulting from this erratic behavior (Koenig et al., 2020; Polson & Scott, 2012). Thus, using these distributions as hyperprior distributions yields more accurate variance estimates; their accuracy in turn plays a crucial role in the accuracy of the item parameter estimates in hierarchical models.

Third, due to its specific parameterization, the OH2PL does not suffer from two problematic dependencies that are common for hierarchical models, especially for small-sample situations (Betancourt & Girolami, 2015): The cross-level dependency of the item parameters **ξ**_*i*_ and their grand means **μ**_**ξ**_ = {μ_α_, μ_β_}, as well as the correlation between the item parameters. Following Koenig et al. (2020), for each item *i*,a vector of uncorrelated *z*-scores $\overset{\sim }{{\boldsymbol{\upxi}}_i}=\left(\overset{\sim }{{\boldsymbol{\upxi}}_1},\dots, \overset{\sim }{{\boldsymbol{\upxi}}_I}\right)$ is drawn from a standard normal distribution. Each individual vector is then multiplied by the diagonal matrix of the variance components **Λ** and the Cholesky factor of the item correlation matrix **L**_**Ω**_ to obtain the item parameters **ξ**_*i*_, that is, ${\boldsymbol{\upxi}}_i={\left(\boldsymbol{\Lambda} {\textbf{L}}_{\boldsymbol{\Omega}}\overset{\sim }{{\boldsymbol{\upxi}}_i}\right)}^{\textrm{T}}$. Two additional transformations, α_*i*_ = exp(μ_α_ + ξ_α*i*_) and β_*i*_ = μ_β_ + ξ_β*i*_, leave only the uncorrelated person parameters θ_*j*_ and a vector of uncorrelated *z*-scores $\overset{\sim }{{\boldsymbol{\upxi}}_i}$ as actively sampled substantial parameters, yielding a joint posterior that is much easier to explore and that results in a more efficient sampling process (Koenig et al., 2020). In the current version of the OH2PL, we introduce an additional optimization related to the hyperpriors for the variance components τ_α_ and τ_β_. With respect to sampling efficiency, the Cauchy distribution may be problematic due to its heavy tails. Thus, instead of sampling the variance components directly, we introduce auxiliary parameters ${\uptau}_{\upalpha_{unif}}$ and ${\uptau}_{\upbeta_{unif}}$ with lower and upper bounds of zero and *π*/2, respectively. We sample these auxiliary parameters from a uniform distribution U(0, *π*/2), and transform them to the actual variance components by ${\uptau}_{\upalpha},{\uptau}_{\upbeta}=2.5\left(\tan \left({\uptau}_{\upalpha_{unif}},{\uptau}_{\upbeta_{unif}}\right)\right)$, where *tan* is the tangent (see also Stan Development Team, 2022). The transformation in this example implies a half − Cauchy(0,2.5) hyperprior on the variance components. A similar transformation is available if we want to use the Exponential distribution. In this case, the aforementioned auxiliary parameters are sampled from a U(0, 1) distribution and transformed into the actual variance components by ${\uptau}_{\upalpha},{\uptau}_{\upbeta}=2.5\left(-\log \left({\uptau}_{\upalpha_{unif}},{\uptau}_{\upbeta_{unif}}\right)\right)$, implying an Exponential(2.5) hyperprior.

Lastly, **L**_**Ω**_ is given a  $\textrm{LKJ}\left({\textbf{L}}_{\boldsymbol{\Omega}}|\upeta \right)=\prod_{k=2}^K{\textrm{L}}_{kk}^{K-k+2\upeta -2}$ prior distribution with shape parameter η > 0, where *k* is the number of dimensions of the *K* × *K* lower triangular Cholesky factor (Lewandowski et al., 2009). As η → ∞, extreme correlations become less likely. The parameter η provides direct control over how closely the sampled matrix resembles the identity matrix (Stan Development Team, 2020). Setting η = 2 results in a weakly informative prior distribution that slightly favors smaller correlations. The prior specifications in (5) and (6) represent specifications for item discrimination and difficulty parameters commonly found in the literature on hierarchical Bayesian IRT models (e.g., Levy & Mislevy, 2016).

## Method

To answer the research question, we conducted a comprehensive simulation study. Models, data, results, and scripts of this study are included in the supplementary material available at the Open Science Framework (OSF) repository 10.17605/osf.io/m3zaq.

### Design

The fully crossed design of the study consisted of the following factors: (1) sample size (*N* = 50, 75, 100, 150, 200, 500), (2) test length (*k* = 25, 50), (3) variance components of the item discrimination and difficulty parameters (τ_α_, τ_β_ = {0.10, 0.40}, {0.25, 0.90}, {0.75, 1.50}), (4) correlation of the item discrimination and item difficulty parameters (ρ_αβ_ = .0, .3), and (5) specifications of the hyperprior distributions for the variance components (half-Cauchy, Exponential, and inverse Gamma distributions). We chose sample sizes and test lengths that mimic testing conditions where accurate item parameter estimates would generally be difficult to obtain (Koenig et al., 2020; Sheng, 2017).

We manipulated the variance components and the correlations to examine the performance of the OH2PL in typical and atypical data conditions. We selected the variance components to reflect small (τ_α_, τ_β_ = {0.10, 0.40}), typical (τ_α_, τ_β_ = {0.25, 0.90}), and extreme (τ_α_, τ_β_ = {0.75, 1.50}) variances in the item parameters. The rationale for choosing these variance levels, and considering them as small, typical, and extreme, is as follows. In operational applications of IRT models for dichotomously scored items, item discrimination and difficulty parameters typically fall in a relatively narrow range. For instance, item discriminations typically fall in the interval [0.5, 3.0], while item difficulties are typically found to be in the interval [−4, 4] (e.g., OECD, 2021). Parameter values outside of these intervals are seldom observed for latent traits with typical variance. This also restricts the variance of the item parameters: for instance, the variance components of the item discrimination and difficulty parameters of the 2018 cycle of the Programme for International Student Assessment (PISA) – a worldwide study to evaluate educational systems and relying on IRT – were mostly smaller than 0.4 and 1.0, respectively (cf. OECD, 2021). Thus, the levels of the variance components connect to item characteristics of operational IRT applications. The selected correlations reflect independent and correlated item parameters. Regarding the hyperprior distributions for the variance components, we focused on weakly informative and noninformative specifications, relative to the generating values for τ_α_ and τ_β_ (see Table 1).

**Table 1:** Specifications of the variance-related hyperprior distributions

Hyperprior	Parameters	Specifications	
Half-Cauchy	Location, Scale	1 – weakly informative I	μ = 0, σ = 1
	μ, σ	2 – weakly informative II	μ = 0, σ = 2.5
		3 – noninformative I	μ = 0, σ = 5
		4 – noninformative II	μ = 0, σ = 25
Exponential	Inverse scale	1 – weakly informative I	*b* = 1
	*b*	2 – weakly informative II	*b* = 0.4
		3 – noninformative I	*b* = 0.2
		4 – noninformative II	*b* = 0.04
Inverse Gamma	Shape, Scale	1 – weakly informative I	*a* = 3, *b* = 2
	*a*, *b*	2 – weakly informative II	*a* = 1, *b* = 0.5
		3 – noninformative I	*a* = 1, *b* = 2
		4 – noninformative II	*a* = 0.001, *b* = 0.001

The rationale for selecting weakly and non-informative prior distribution follows Gelman (2009) who argues that regularization of parameters is necessary when working with complex hierarchical models in small sample situations. Weakly informative prior distributions strike a balance between unwarranted influences on the posterior distribution, while at the same time avoiding unrealistic parameter values that may have detrimental effects on the sampling behavior and unwanted effects on the posterior (which may be the case for non-informative prior distributions).

The different specifications of the prior distributions under investigation differ in their range of uncertainty around the plausible values of the variance components (Gelman & Hill, 2007); this range of uncertainty increases when moving from the weakly to the non-informative specifications considered in this simulation. We aimed at including a relatively broad range of possible specifications. The choice of their specific hyperparameters is based on previous comparisons, recommendations and use-cases found in the methodological literature (e.g., Bürkner, 2021; Koenig et al., 2020; Luo & Jiao, 2018; Natesan et al., 2016; Röver et al., 2021; Sheng, 2017), with a focus on the comparability of the resulting densities. Please note that due to its low mass near zero, it is inherently difficult to specify inverse Gamma distributions that are fully comparable to the half-Cauchy and Exponential distributions. We included, however, specifications that are approximately comparable and that are used in methodological and empirical studies (e.g., Depaoli et al., 2021; Gardini et al., 2021; Koenig et al., 2020; Matzke et al., 2018; Sheng, 2017; Smid & Rosseel, 2020; Smid & Winter, 2020). Lastly, we did not focus exclusively on the commonly used highly noninformative specifications of the inverse Gamma distribution, because they are already known to be problematic (Gelman, 2006; Röver et al., 2021). Illustrations of the different densities are included in the electronic supplementary material in the OSF respository (Supplement 1).

In total, 864 conditions were examined across the five simulation factors. To provide a complete picture of the robustness of the OH2PL, we compared its performance with its nonhierarchical counterpart, its more common inverse Wishart specification (e.g., Levy & Mislevy, 2016), and with MML estimation, which is implemented in most modern non-Bayesian IRT modeling software packages.

### Data generation and analysis

The generation of data aimed to yield parameter values typically found in operational tests based on the unidimensional 2PL model and to avoid unrealistic item discriminations and item difficulties. We used the following procedure to achieve this.

First, for each item *i*, a parameter vector **ξ**_*i*_ was drawn from a truncated bivariate normal distribution with grand mean vector **μ**_**ξ**_ ***=*** {1, 0}, lower limits *LL* = {0.65, −4.5} and upper limits *UL* = {4.0,4.5}, and diagonal matrix **τ**, resulting in the auxiliary matrix **Z**. Second, the parameter matrix **Ξ** with the desired variances and correlations was obtained by $\boldsymbol{\Xi} =\textbf{S}\ {\textbf{L}}_{\textbf{S}}^{-\textbf{1}}\ {\textbf{L}}_{\boldsymbol{\Sigma}}$, where **S** is the covariance matrix of **Z**, ${\textbf{L}}_{\textbf{S}}^{-\textbf{1}}$ is the inverse of the Cholesky factor of **S**, and **L**_**Σ**_ is the Cholesky factor of the population covariance matrix **Σ** = **τ Ω τ**^T^, with **τ** being a diagonal matrix of the variance components and **Ω** being the population correlation matrix. Lastly, the generating item parameters were obtained by mean centering each column of **Ξ** and adding the true (marginal) means of the truncated bivariate normal distribution, which ensured that the grand means of the item parameters **μ**_**ξ**_ were correct. This procedure generated item parameters in the ranges of 0.5 < α_*i*_ < 3.5 and −4 < β_*i*_ < 4 with the desired grand means, variances, and correlations. Person parameters were drawn from a standard normal distribution θ_*j*_~N(0, 1), yielding a 99% CI [−3.11, 3.11]. For each of the 864 simulation conditions, 100 data sets were generated. Different sets of item and person parameters were drawn for each data set.

The standard inverse Wishart H2PL was specified with θ_*j*_~N(0, 1), **ξ**_*i*_~MVN(**μ**_**ξ**_, **Σ**), μ_α_~N(0, 1), μ_β_~N(0, 2), and **Σ**~IW(3, **I**), where **I** is the identity matrix. The nonhierarchical 2PL was specified with θ_*j*_~N(0, 1), α_*i*_~logN(0, 1), and β_*i*_~N(0, 2). These prior configurations are widely used in Bayesian IRT modeling, and represent common ways to specify the 2PL hierarchically and nonhierarchically (e.g., Fox, 2010; Levy & Mislevy, 2016).

To estimate the Bayesian models, Stan (Carpenter et al., 2017) and its R interface *RStan* were used (see Jiang & Carter, 2019, for benefits of Stan other than flexibility in prior specification). Three chains, each 4000 iterations long, with 1000 burn-in cycles were set up. Different random starting values were supplied to each chain. Convergence was assessed using the Gelman–Rubin *R*-statistic (Gelman & Rubin, 1992), where *R* < 1.05 indicated convergence. There was no systematic clustering of non-convergent solutions (non-convergence rate smaller than ten percent) except for the second non-informative specification of the half-Cauchy distribution (NI2, cf. Table 1). Here, the non-convergence rate ranged between 18% and 36% across all design factors (sample size, test length, correlation, and variance components). This illustrates the theoretically expected problem of the half-Cauchy distribution: in non-informative specifications, it allows for very large values; thus, it is more likely for the sampler to be stuck in regions with low probability mass. This leads to sampling inefficiencies and convergence issues. In contrast, both the Exponential and the inverse Gamma distribution yield estimates that are more conservative. The higher convergence rate of these distributions, even in non-informative specifications, reflects this behavior. For the MML estimation of the model, the R package *mirt* was used (Chalmers, 2012). To compare the resulting item parameter estimates, they were transformed from slope/intercept to classical IRT parameterization. Here, inadmissible solutions systematically clustered in the extreme variance conditions. Here, the rate ranged between 10% and 99%; the proportion of inadmissible solutions was highest in case of a sample size of *N* = 50 for both test lengths (range between 75% and 89%). Additionally, MML estimation produced a number of solutions that apparently converged but produced negative item discriminations. These solutions clustered in conditions with small sample sizes (*N* < 100); their proportion ranged between 11% and 25%). Non-convergent (in case of the Bayesian models) and inadmissible (in case of MML estimation) were discarded and not used for the calculation of the results.

### Evaluation criteria

To assess the performance of the OH2PL we used the bias and the root mean square error (*RMSE*) of the estimates of the variance components, the item parameters, and the person parameters; smaller bias and *RMSE* values indicated better performance. We calculated the bias as *B* = π_est_ − π_true_, and the *RMSE* as $RMSE=\sqrt{\sum_{\textrm{R}}{\left({\uppi}_{\textrm{est}}-{\uppi}_{\textrm{true}}\right)}^2/R}$, with π_est_ being the estimated value of a parameter, π_true_ the true value of a parameter, and *R* the number of replications. For item parameters, we averaged the bias across items in each replication. We averaged these averaged indices and the bias in the variance components across replications. We considered the performance of the OH2PL robust if the conditional bias and *RMSE* do not distinctively differ across the specifications of the respective hyperprior distribution. Therefore, for a single simulation condition, we calculated the conditional mean (averaged across hyperprior distributions) of the average bias and *RMSE* and assessed whether the average bias and *RMSE* produced by the individual hyperprior specifications significantly differed from the conditional mean.

## Results

### Performance of the OH2PL is robust for small and typical variances τ_α_, τ_β_

**Fig. 1:**
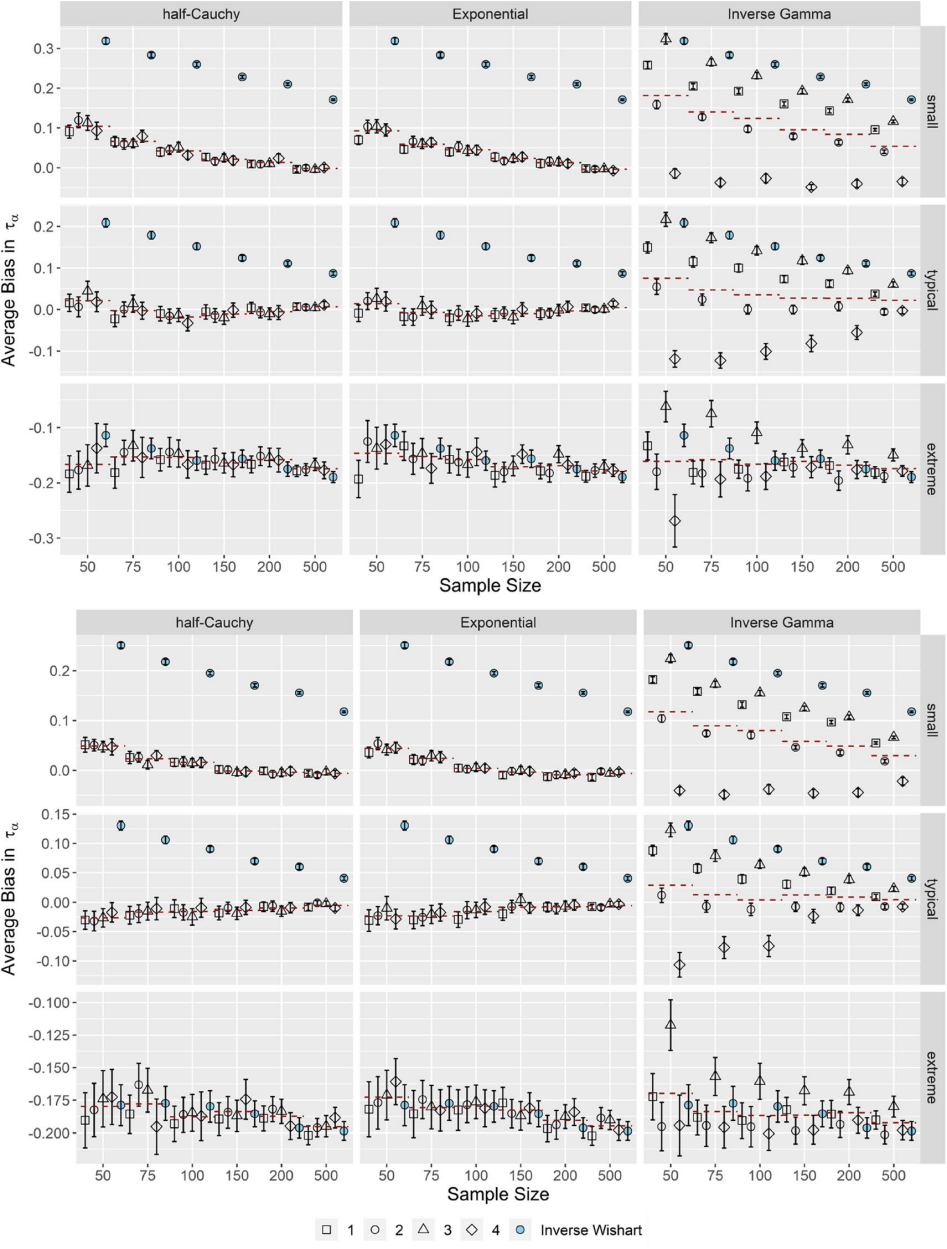
Average bias in τ_α_ for correlated item parameters. *Note.* Upper half *k* = 25 items (short), lower half *k* = 50 items (long). Specifications of the hyperprior distributions: 1 = weakly informative I, 2 = weakly informative II, 3 = noninformative I, 4 = noninformative II (see Table 1 ). Dashed red lines indicate the conditional average bias, averaged across prior specifications. Error bars indicate ± 2 *SE*

**Fig. 2:**
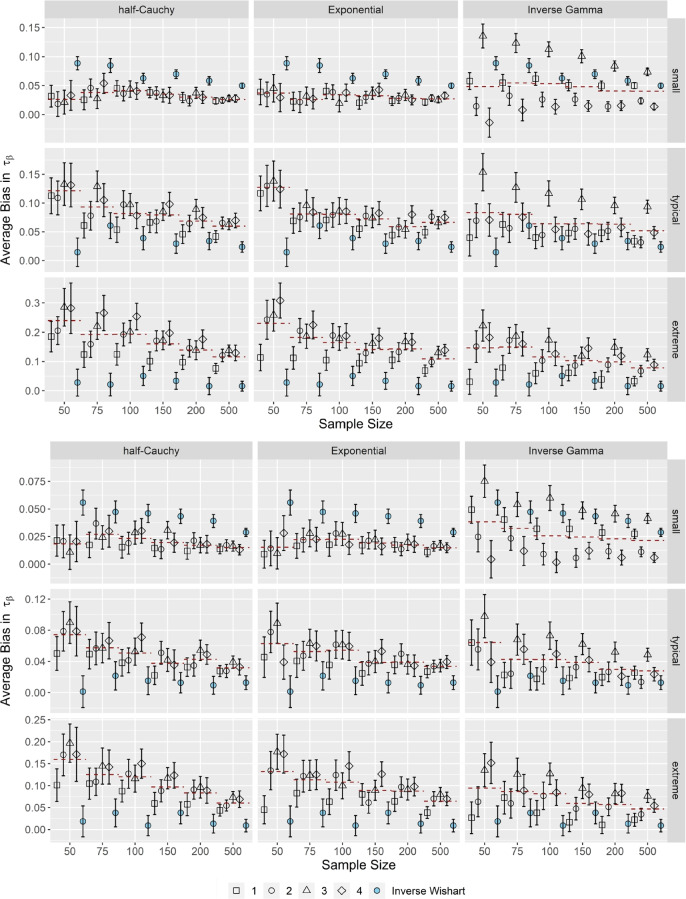
Average bias in τ_β_ for correlated item parameters. *Note*. Upper half *k* = 25 items (short); lower half *k* = 50 items (long). Specifications of the hyperprior distributions: 1 = weakly informative I, 2 = weakly informative II, 3 = noninformative I, 4 = noninformative II (see Table 1 ). Dashed Dashed red lines indicate the conditional average bias, averaged across prior specifications. Error bars indicate ± 2*SE*

Figures 1 and 2 illustrate the average bias in the variance components *τ*_*α*_ and *τ*_*β*_ across small, typical, and extreme variances of the item parameters for correlated item parameters and short (upper half) and long (lower half) test lengths. Correlations of the item parameters did not have a substantial impact on the performance of the OH2PL with regard to the average bias of the variance components; the results for independent item parameters are included in the electronic supplementary material in the OSF repository (Supplement 2).

Figure 1 shows that the OH2PL was robust against specifications of its variance-related hyperprior distributions if either the half-Cauchy or Exponential distributions were used, especially when the true variance was small or typical. It made no difference whether weakly informative or non-informative specifications were used. In contrast, in case of the inverse Gamma distribution, the average bias in the item discriminations was highly dependent of the specification of the prior. In case of small and typical variances, the average bias remained below 0.1 across all sample sizes for the half-Cauchy and Exponential distributions. The variance component τ_α_ was marginally overestimated when sample sizes were smaller than *N* = 100. In contrast, when the inverse Gamma distribution was used, we observed that the average bias ranged between –0.1 and 0.3, depending on the specification of the prior distribution.

In case of extreme variances, the variance component τ_α_ was underestimated regardless of which distribution was used. The average bias remained largely independent of specification in case of the half-Cauchy and Exponential distributions when *N* ≥ 100; here, the most informative specification was associated with the largest amount of average bias. The dependency related to the inverse Gamma distribution reduced, but it was still more distinct compared to the half-Cauchy and Exponential distributions.

Moreover, the OH2PL performed better in terms of average bias (it was consistently smaller across simulation conditions) than the standard inverse Wishart specification of the H2PL, especially when either the half-Cauchy or the Exponential distribution was used. The differences between the OH2PL specifications and the standard inverse Wishart specification of the H2PL disappeared, however, when the variance τ_α_ was extreme.

This pattern of results was similar for both test lengths. As illustrated in Fig. 1, the difference in average bias between short and long tests was negligible.

As shown in Fig. 2, when the true variance τ_β_ was small, the pattern of results was similar to small and typical τ_α_: the OH2PL was robust against the specification of its hyperprior distribution when the half-Cauchy or the Exponential distribution was used. The average bias remained below 0.05, regardless of specification used. It was sensitive, however, to the specification of the inverse Gamma distribution, where the average bias depended on the specification of the prior distribution. Moreover, the half-Cauchy and Exponential distributions consistently performed better than the standard inverse Wishart specification of the H2PL. Estimates of τ_β_ exhibited smaller average bias than the standard inverse Wishart specification of the H2PL across sample sizes, test lengths and correlations in the case of the half-Cauchy and Exponential specifications.

When the true variance τ_β_ increased to typical values, the differences between the half-Cauchy, Exponential, and inverse Gamma specifications decreased: average bias was marginally dependent of the specification of both the half-Cauchy and Exponential distributions, where the most informative specification yielded the least amount of bias. Most specifications of the inverse Gamma distribution yielded an average bias comparable to the alternative distributions, except for the first non-informative distribution, which was associated with the largest amount of average bias. In this condition, estimates of τ_β_ were moderately overestimated (the average bias remained below 0.1).

In case of extreme τ_β_, the performance of the inverse Gamma distribution improved further and was similar to the performance of the half-Cauchy and Exponential distributions. The average bias depended on the specification for all three hyperprior distributions, and the variance component was overestimated, especially in sample sizes smaller than *N* < 100. In general, the most informative specification yielded the least amount of bias. Lastly, estimates of τ_β_ obtained by the standard inverse Wishart specification of the H2PL exhibited smaller average bias compared to the half-Cauchy and Exponential specifications of the OH2PL across sample sizes and test lengths.

Overall, the OH2PL was robust in terms of average bias for small and typical τ_α_. For extreme τ_α_, estimates were moderately underestimated. Similarly, for small τ_β_, the OH2PL was robust in terms of average bias. In these situations, the average bias was independent from the specification of the half-Cauchy and Exponential distributions, and highly dependent from the specification of the inverse Gamma distribution. When τ_β_ increased, the amount of bias increased to the moderate range, and the hyperprior distributions performed similarly. The standard inverse Wishart specification of the H2PL performed worse than the OH2PL, especially with the half-Cauchy and Exponential configurations, except for extreme τ_α_ and typical/extreme τ_β_. In general, average bias decreased slightly with an increasing test length.

### The OH2PL is robust in terms of the accuracy of the item parameters α, β

**Fig. 3:**
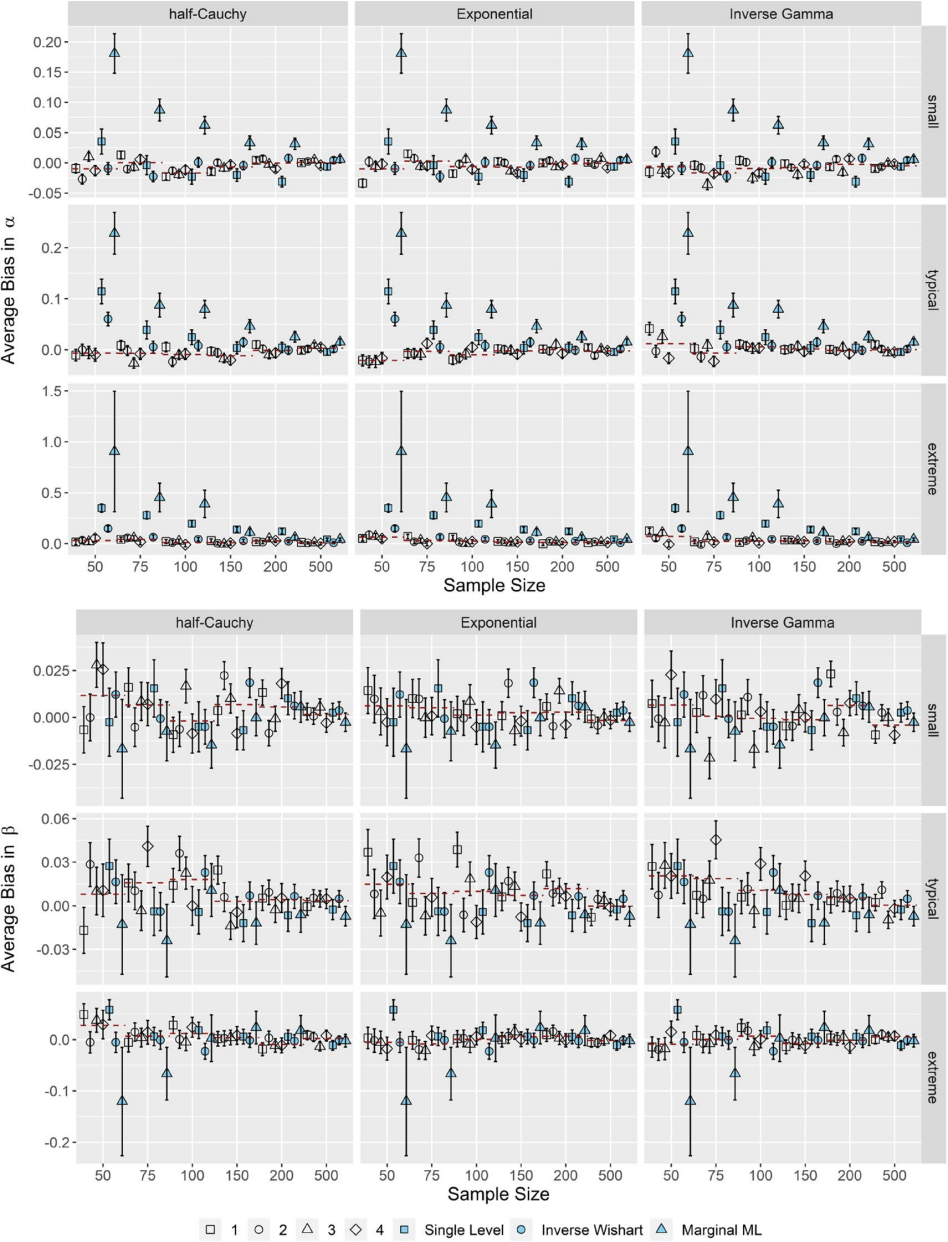
Average bias in the item discrimination and difficulty parameters α, β. * Note*. Results for correlated item parameters and *k* = 25. Specifications of the hyperprior distributions: 1 = weakly informative I, 2 = weakly informative II, 3 = noninformative I, 4 = noninformative II (see Table 1). Dashed red lines indicate the conditional average bias, averaged across prior specifications. Error bars indicate ± 2 *SE*

Figure 3 shows the average bias in the item discrimination parameter α and item difficulty parameter β for small, typical, and extreme variances of both item parameters. Because test length and correlation did not have a substantial impact on average bias (the pattern of results was virtually the same across these conditions), Fig. 3 illustrates results for correlated item parameters and short test lengths only (all complementary results are included in the electronic supplementary material in the OSF repository; Supplement 2).

The results showed that the OH2PL yielded unbiased estimates of the item discrimination parameters α: with the exception of the inverse Gamma configuration for *N* = 50 and extreme τ_α_, average bias was close to zero across all simulation conditions. Hence, the negligible bias was independent of the specification of the hyperprior distributions and sample size. Thus, we considered the OH2PL in its half-Cauchy or Exponential configuration robust in terms of the accuracy of the item discrimination parameters α. Moreover, the OH2PL with either the half-Cauchy or the Exponential configuration showed considerable advantages in comparison to MML estimation in sample sizes smaller than *N* < 150 across all simulation conditions (the larger number of inadmissible solutions in the respective conditions could explain the increased standard errors of MML estimation). For typical and extreme τ_α_, the performance of the OH2PL was slightly better than the standard inverse Wishart specification of the H2PL and its nonhierarchical counterpart; there were no distinct differences in average bias for small variances.

There were no differences in the average bias of the item difficulty parameters β between the OH2PL configurations (see the lower half of Fig. 3), the standard inverse Wishart specification of the H2PL, its non-hierarchical counterpart, and MML estimation. The amount of bias was near zero (well within ± 0.04) across all simulation conditions for small, typical, and extreme variances τ_β_. Thus, we considered the OH2PL robust in terms of the accuracy of the item difficulty parameters β.

Overall, the OH2PL was robust in terms of the accuracy of the item parameters α, β, especially with the half-Cauchy or exponential configuration. In general, average bias in α increased slightly with increasing variances τ_α_, τ_β_, while the average bias in β did not.

### The OH2PL is robust in terms of the accuracy of the person parameter θ

**Fig. 4:**
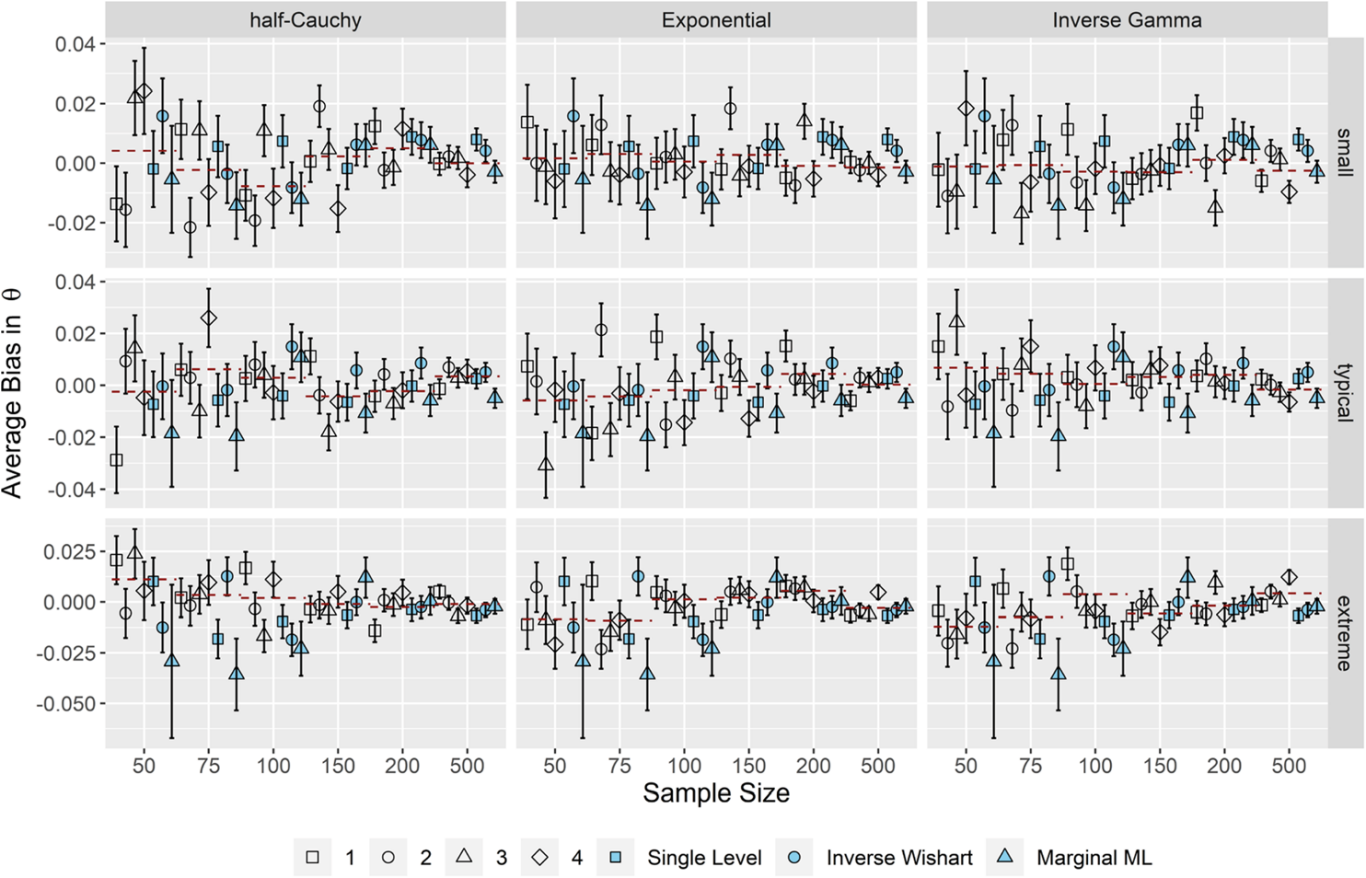
Average bias in the person parameters θ. *Note*. Results for correlated item parameters and *k* = 25. Specifications of the hyperprior distributions: 1 = weakly informative I, 2 = weakly informative II, 3 = noninformative I, 4 = noninformative II (see Table 1). Dashed red lines indicate the conditional average bias, averaged across prior specifications. Error bars indicate ± 2 *SE*

Figure 4 illustrates the average bias in the person parameters θ for correlated item parameters and *k* = 25 (results for *k* = 50 and independent item parameters were indistinguishable; they are included in the electronic supplementary material in the OSF repository; Supplement 2).

Overall, we considered the OH2PL robust in terms of the accuracy of the person parameters θ. The average bias remained between ± 0.03 across all simulation conditions and specifications of the hyperprior distributions in question. Furthermore, the average bias was largely independent of sample size. Again, the larger number of inadmissible solutions in the respective conditions could explain the increased standard errors of MML estimation. Moreover, differences between the OH2PL configurations, the standard inverse Wishart specification of the H2PL, its nonhierarchical counterpart, and MML estimation were negligible.

### Superior performance of the OH2PL for small and typical τ_α_, τ_β_ in smallest samples

**Fig. 5:**
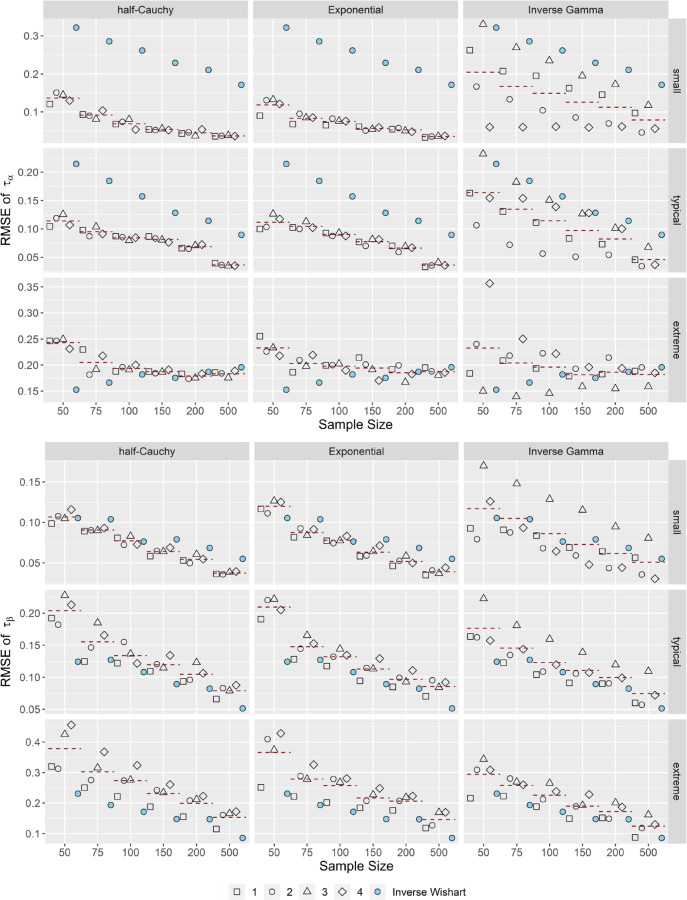
*RMSE* of the estimated variance components τ_α_ and τ_β_. *Note*. Results for correlated item parameters and *k* = 25. Specifications of the hyperprior distributions: 1 = weakly informative I, 2 = weakly informative II, 3 = noninformative I, 4 = noninformative II (see Table 1). Dashed red lines indicate the conditional average bias, averaged across prior specifications

Figure 5 illustrates the *RMSE* in the variance components τ_α_, τ_β_ for correlated item parameters and *k* = 25. Compared to the standard inverse Wishart specification of the H2PL, the OH2PL, especially in its half-Cauchy or Exponential configuration, exhibited a superior performance when τ_α_ was small or typical, and when τ_β_ was small. Moreover, the superior performance was independent of the specification of the half-Cauchy or Exponential distribution. Although, from a strict statistical point of view, there were differences in *RMSE* between the specifications, these differences were too small to be practically relevant. When τ_α_ was extreme, the standard inverse Wishart specification of the H2PL performed slightly better in the smallest sample sizes (*N* < 100). When τ_β_ was typical or extreme, the standard inverse Wishart specification of the H2PL performed better across all sample sizes. Differences to the inverse Gamma configuration of the OH2PL were not that distinct, although the former remained highly sensitive to its specification. Increasing the test length (not shown) lead to a decrease in *RMSE* of around 0.05 across all model specifications. Correlation did not have an impact on the *RMSE* of the estimated variance components.

**Fig. 6:**
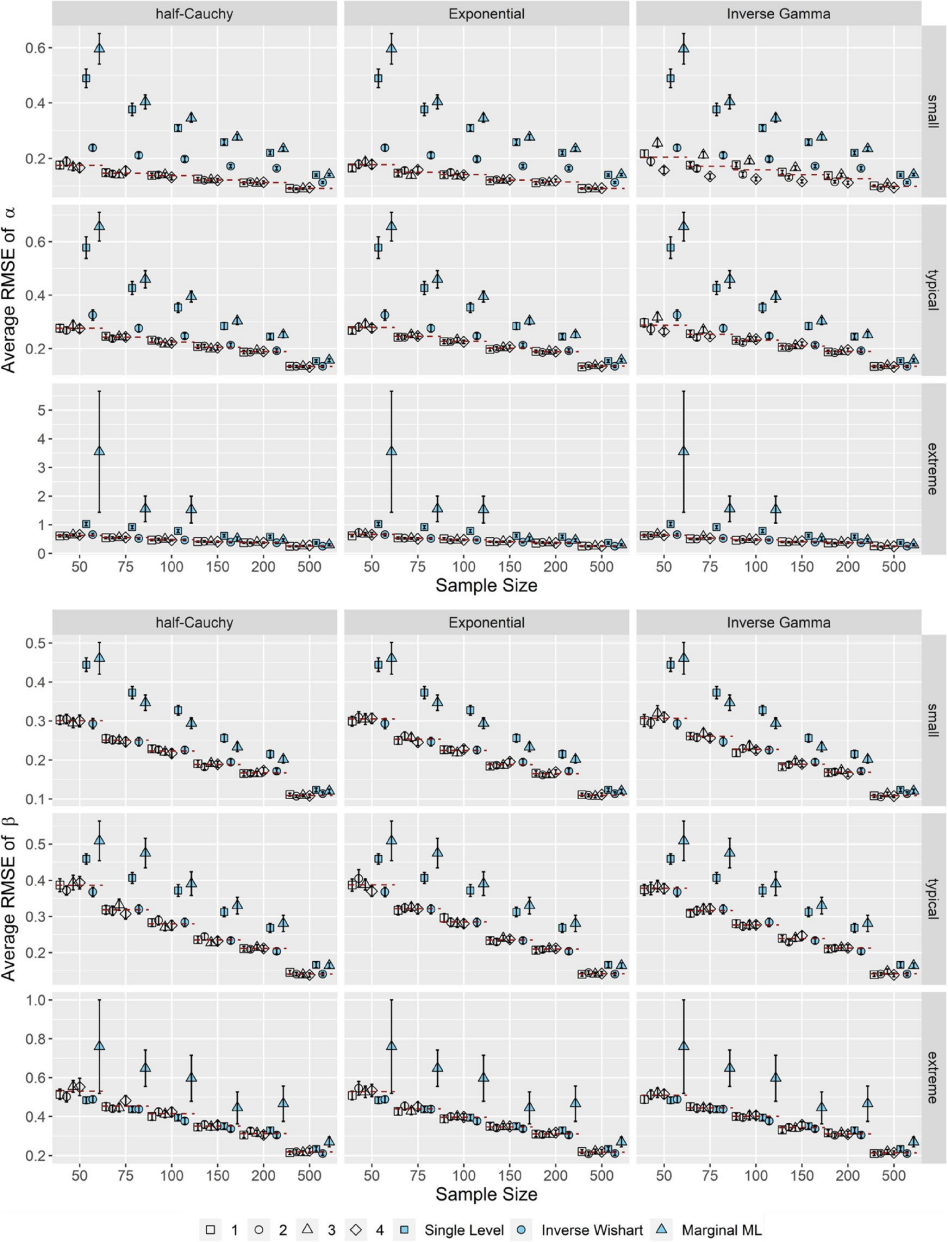
Average *RMSE* of the item discrimination and difficulty parameters α, β. *Note*. Results for correlated item parameters and *k* = 25. Specifications of the hyperprior distributions: 1 = weakly informative I, 2 = weakly informative II, 3 = noninformative I, 4 = noninformative II (see Table 1). Dashed red lines indicate the conditional average bias, averaged across prior specifications. Error bars indicate ± 2 *SE*

The upper half of Fig. 6 shows (for correlated item parameters and *k* = 25) that in case of small τ_α_, the OH2PL, regardless of its configuration, exhibited a superior performance in terms of the average *RMSE* of the item discrimination parameters α, compared to the standard inverse Wishart specification of the H2PL, its nonhierarchical counterpart and to MML estimation across simulation conditions, except for *N* = 500. The advantages of the OH2PL compared to the standard inverse Wishart specification of the H2PL decreased as τ_α_, τ_β_ increased. Regarding the item difficulty parameters β, the OH2PL and the standard inverse Wishart specification of the H2PL showed a superior performance compared to their nonhierarchical counterpart and MML estimation for sample sizes *N* < 500 (see the lower half of Fig. 6). The advantage compared to its nonhierarchical counterpart decreased when τ_α_, τ_β_ were extreme. Moreover, between the OH2PL and the standard inverse Wishart specification of the H2PL, there were no differences in the average *RMSE* of the item difficulty parameters. For both item parameters, the performance of the OH2PL in terms of *RMSE* was independent of the specific specification of its variance-related hyperprior distribution. It has to be noted, however, that the performance of the inverse Gamma distribution in case of the item discriminations, especially when the true variance is small, was relatively sensitive to its specification. Neither test length nor correlation had a distinct impact on the *RMSE* of the estimated item parameters. Lastly, as can be seen by the inflated standard errors of the *RMSE* estimates, the MML estimation had severe convergence issues in smaller sample sizes and when the true variance components of the item parameters were extreme.

**Fig. 7:**
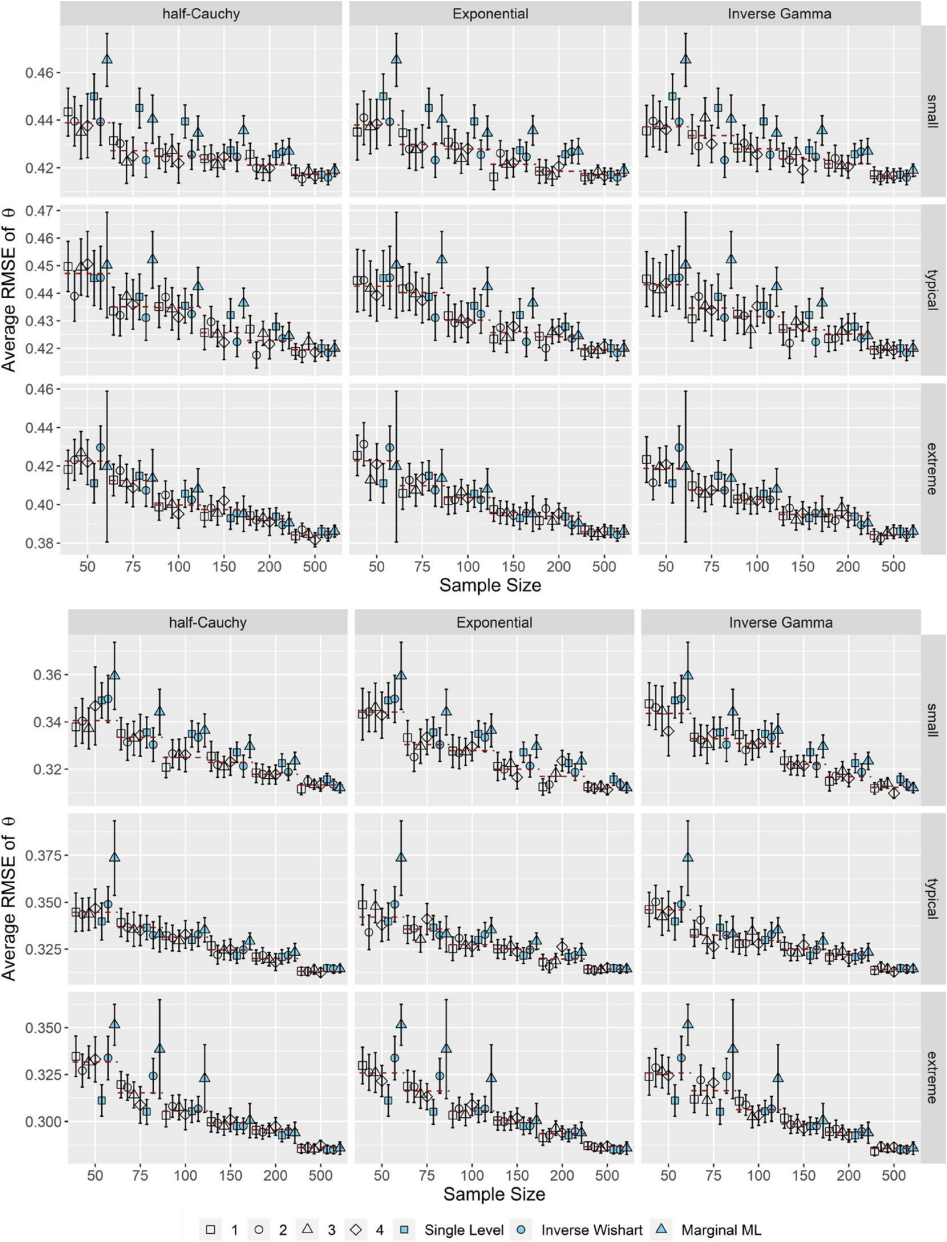
Average *RMSE* of the person parameters θ for correlated item parameters. *Note*. Upper half: *k* = 25 (short), lower half *k* = 50 (long). Specifications of the hyperprior distributions: 1 = weakly informative I, 2 = weakly informative II, 3 = noninformative I, 4 = noninformative II (see Table 1). Dashed red lines indicate the conditional average bias, averaged across prior specifications. Error bars indicate ± 2 *SE*

Lastly, as shown in Fig. 7, there were only marginal differences between MML estimation and the OH2PL regarding the average *RMSE* of the person parameters θ when τ_α_, τ_β_ were either small or typical. Moreover, the average *RMSE* decreased with increasing test length. Differences in the performance of the OH2PL, its nonhierarchical counterpart, and the standard inverse Wishart specification of the H2PL were negligible. The performance of the OH2PL in terms of *RMSE* of the person parameters θ was independent of the specification of its hyperprior distribution, across all simulation conditions and distributions. Increasing the test length (not shown) lead to a small decrease in *RMSE* across all model specifications. Correlation did not have an impact on the *RMSE* of the estimated person parameters θ.

Overall, the OH2PL in its half-Cauchy or Exponential configuration showed a similar or superior performance in terms of the *RMSE* of the variance components (compared to the standard inverse Wishart specification of the H2PL), of the item parameters α, β, and (partly) of the person parameters θ, compared to the nonhierarchical counterpart and MML estimation.

Results of two supplementary simulations corroborated this finding. These simulations focused on two specific aspects. First, we wanted to investigate the performance of the OH2PL relative to the other model specifications when the average item difficulties did not match the average person ability. This is a common situation in psychological research and may have an impact on model performance. Overall, the results of the first supplementary simulation showed that shifts in the difficulty distribution against the ability distribution had no impact on the performance of the different configurations of the OH2PL. Second, we aimed at providing further insights by investigating the performance of the OH2PL relative to the other model specifications when following a fixed item parameter approach, with a special focus on the item discrimination parameter. Overall, the results of the second supplementary simulation showed that the OH2PL provided item discrimination estimates that were largely unbiased across the whole range of possible discrimination values when the sample size was at least *N* = 100. Detailed results are available in the electronic supplementary material in the OSF repository (Supplement 1).

### Empirical example

To illustrate the increased applicability of the 2PL IRT model when using the Bayesian hierarchical approach, we used empirical response data from a university exam. This exam tested in how far the students have acquired skills and knowledge covered by a lecture on the fundamentals of empirical research methods in psychology within the psychology bachelor’s program at a German university. The exam consisted of *I* = 27 items administered to *N* = 80 students in the summer term 2021.

Thus, the number of students was well below the sample size recommended for estimating the 2PL IRT model. Moreover, in fact, the exam data had been calibrated using the Rasch model so far, even though this meant limiting the amount of information compared to IRT models with discrimination parameters. The average difficulty of the items was $\overline{\upbeta}=-2.13$, and three items had a probability of a correct response higher than 90% (there was one item that every student answered correctly). In sum, it was a relatively easy exam, but from an estimation point of view, it nicely represented suboptimal testing conditions and therefore was a very good situation to exemplify the potential of the OH2PL.

We compared the item parameters obtained from the OH2PL in its second weakly informative Exponential configuration (*τ*_*α*, *β *_~ *Exp*(0.4)) to the item parameters obtained from the inverse Wishart specification, its Bayesian non-hierarchical counterpart, and obtained from MML estimation. Neither the hierarchical nor the non-hierarchical estimates showed any convergence issues: the Gelman-Rubin *R*-statistics of all parameters were close to one, and their effective sample sizes were well over 400 (Zitzmann & Hecht, 2019). There were serious convergence issues with MML estimation, however. We had to change the optimizer and to introduce additional information via a lognormal prior for the discrimination parameter of the first item to avoid a negative discrimination and to achieve convergence.

Table 2 illustrates the item parameters obtained with the three approaches. It is obvious that, although it converged, the MML solution was not admissible mostly because of the response patterns at the boundary (the number of near perfect response patterns was too large). The average discrimination of the OH2PL was $\overline{\upalpha}=1.29$ (*SD* = 0.77), of the inverse Wishart specification $\overline{\upalpha}=1.27$ (*SD* = 0.71), and of the non-hierarchical 2PL $\overline{\upalpha}=1.52$ (*SD* = 1.24). The average difficulty of the OH2PL was $\overline{\upbeta}=-2.13$ (*SD* = 0.45), of the inverse Wishart specification $\overline{\upbeta}=-2.12$ (*SD* = 0.45), and of the non-hierarchical 2PL $\overline{\upbeta}=-1.95$ (*SD* = 0.78). Especially the pattern regarding the discrimination was quite similar to the results of our simulation for the extreme variance component. Thus, it was possible that the average item discrimination of the non-hierarchical 2PL was inflated due to the items with extreme response patterns; the OH2PL (and the inverse Wishart specification) were able to avoid this inflation due to shrinkage. Also, note that the average item difficulty of the OH2PL model matched the average difficulty of the Rasch-calibrated items. In sum, this empirical example illustrated how the OH2PL extended the applicability of the 2PL IRT model to suboptimal testing conditions: together with the inverse Wishart specification, it was better able to handle extreme response patterns, which are more likely in smaller samples, by shrinking extreme parameter estimates towards their grand means, thus avoiding inflated estimates. Hence, test administrators can keep certain items in an item bank after initial calibration and may recalibrate them as new response data becomes available.

**Table 2:** Estimated item parameters of the empirical example

Item	Discrimination								Difficulty							
	OH2PL		IW		Single		MML		OH2PL		IW		Single		MML	
	Est	*SE*	Est	*SE*	Est	*SE*	Est	*SE*	Est	*SE*	Est	*SE*	Est	*SE*	Est	*SE*
1	0.36	0.13	0.36	0.13	0.28	0.13	**0.39**	**0.49**	– 1.97	0.71	– 1.94	0.68	– 2.03	1.03	– 1.82	2.24
2	1.64	0.60	1.62	0.56	1.73	0.76	1.77	0.83	– 2.36	0.54	– 2.36	0.49	– 2.20	0.73	– 2.15	0.62
3	3.15	2.28	2.95	1.89	4.37	3.70	**49.02**	**210.11**	– 2.66	0.61	– 2.66	0.55	– 2.40	0.71	– 2.10	0.43
4	0.85	0.26	0.85	0.25	0.80	0.31	0.61	0.41	– 2.26	0.59	– 2.23	0.54	– 2.31	0.87	– 2.89	1.76
5	1.41	0.53	1.39	0.50	1.81	0.80	2.18	0.87	– 2.10	0.53	– 2.09	0.48	– 1.65	0.55	– 1.55	0.35
6	3.28	3.49	3.04	1.79	3.77	3.23	***NA***	***NA***	– 3.03	0.72	– 3.02	0.61	– 3.51	1.04	***NA***	***NA***
7	1.95	0.84	1.89	0.65	1.65	0.73	0.51	1.24	– 2.90	0.69	– 2.88	0.56	– 3.34	1.02	**– 8.83**	**20.43**
8	0.65	0.30	0.66	0.31	0.86	0.38	0.87	0.37	– 1.26	0.60	– 1.25	0.61	– 0.57	0.49	– 0.63	0.37
9	1.33	0.43	1.33	0.42	1.28	0.52	1.23	0.63	– 2.44	0.57	– 2.43	0.52	– 2.45	0.80	– 2.50	0.94
10	2.86	2.30	2.73	2.00	5.79	4.20	**42.72**	**277.65**	– 1.76	0.43	– 1.76	0.42	– 1.18	0.31	– 1.23	0.23
11	0.57	0.21	0.58	0.20	0.64	0.28	0.62	0.34	– 1.74	0.58	– 1.72	0.58	– 1.34	0.74	– 1.39	0.76
12	1.07	0.45	1.09	0.46	1.53	0.59	1.49	0.55	– 1.47	0.48	– 1.45	0.47	– 0.88	0.40	– 0.98	0.31
13	0.65	0.20	0.65	0.19	0.57	0.22	0.27	0.34	– 2.22	0.60	– 2.20	0.57	– 2.39	0.90	– 4.89	6.07
14	1.04	0.36	1.04	0.36	1.20	0.48	1.35	0.55	– 1.97	0.52	– 1.95	0.50	– 1.61	0.64	– 1.51	0.46
15	1.87	0.81	1.86	0.77	2.17	1.10	1.98	0.93	– 2.26	0.51	– 2.25	0.49	– 1.96	0.61	– 2.03	0.56
16	0.84	0.27	0.85	0.27	0.90	0.35	0.87	0.41	– 2.01	0.54	– 2.00	0.52	– 1.79	0.72	– 1.83	0.74
17	0.89	0.27	0.89	0.26	0.87	0.34	0.79	0.45	– 2.29	0.59	– 2.28	0.53	– 2.30	0.84	– 2.45	1.21
18	0.95	0.32	0.95	0.32	1.12	0.46	1.30	0.51	– 1.91	0.53	– 1.90	0.50	– 1.52	0.65	– 1.40	0.43
19	1.15	0.38	1.15	0.38	1.23	0.52	1.22	0.54	– 2.12	0.52	– 2.09	0.49	– 1.89	0.71	– 1.89	0.64
20	1.42	0.50	1.41	0.48	1.52	0.67	1.56	0.71	– 2.27	0.53	– 2.27	0.49	– 2.07	0.70	– 2.03	0.61
21	1.34	0.42	1.32	0.39	1.07	0.40	**0.03**	**0.72**	– 2.84	0.68	– 2.82	0.57	– 3.38	1.01	**– 98.75**	**2169.64**
22	1.28	0.42	1.27	0.41	1.29	0.53	1.27	0.61	– 2.28	0.55	– 2.26	0.50	– 2.16	0.74	– 2.17	0.76
23	0.41	0.18	0.42	0.18	0.42	0.21	0.36	0.30	– 1.59	0.68	– 1.56	0.67	– 1.06	0.85	– 1.31	1.19
24	1.39	0.46	1.38	0.44	1.27	0.52	1.08	0.63	– 2.53	0.61	– 2.51	0.53	– 2.65	0.88	– 2.93	1.34
25	0.47	0.21	0.48	0.22	0.58	0.28	0.73	0.34	– 1.37	0.67	– 1.35	0.66	– 0.59	0.63	– 0.56	0.41
26	0.89	0.33	0.90	0.33	1.14	0.45	1.20	0.45	– 1.69	0.53	– 1.67	0.50	– 1.18	0.54	– 1.18	0.39
27	1.11	0.35	1.11	0.34	1.10	0.43	1.04	0.52	– 2.31	0.55	– 2.31	0.53	– 2.26	0.80	– 2.35	0.93

*Note*. Item 6 could not be estimated with MML due to a perfect response pattern (all correct). Problematic estimates in bold

## Discussion

The primary purpose of this study was to investigate the robustness of the performance of the OH2PL in terms of parameter accuracy in calibration sample sizes below 500 respondents. Therefore, we focused primarily on differences in performance due to different specifications of the hyperprior structure of the OH2PL and investigated this performance across a broad range of data conditions. We illustrated the advantages of the OH2PL in an empirical example.

The OH2PL in its half-Cauchy and Exponential configurations outperformed the standard inverse Wishart specification of the H2PL with regard to the bias and accuracy of the variance components for small and typical τ_α_, and for small τ_β_. Moreover, in contrast to the inverse Gamma configuration, the half-Cauchy and Exponential configurations were consistent, that is, their performance did not depend on the specification of the hyperprior distribution. This supports and complements Gelman (2006), Polson and Scott (2012), and more recently Sheng (2017), who all recommended distributions from the half-*t* family as reasonable hyperprior distributions for variance components in hierarchical Bayesian (IRT) models. Using these distributions resolves the restrictive nature of the inverse Gamma distribution near zero by allowing either small or large variance components. The smaller advantage of the half-Cauchy or Exponential configuration of the OH2PL in the case of extreme variance components (and τ_β_ in general) can be explained by the larger variances of the item discrimination and difficulty parameters in these conditions. Because the inverse Gamma distribution is problematic especially in cases when the variance is near zero (Gelman, 2006), the larger variances allow this distribution to perform better. The similar or better performance of the inverse Gamma distribution observed for extreme variances, and τ_β_ in general, is expected; when the variance component increases and moves away from zero, it moves into the highest density regions of the inverse Gamma distribution, and away from regions with very low probability mass. Therefore, the bias due to the true value lying outside the highest density region of the inverse Gamma distribution should be smaller. This essentially implies that if the typical value of a variance component is known to be sufficiently large, there is nothing against using the inverse Gamma distribution as hyperprior for the variance components, potentially helping in case of convergence problems with the Cauchy/Exponential distributions. Nevertheless, it remains sensitive to its specification, albeit to a lesser extent than in case of small or typical variances.

Moreover, our results show that when the variance components of the item parameters are large (more specifically, extreme in case of the item discriminations and typical/extreme in case of the item difficulties), the inverse Wishart distribution performs similar and even better than the alternative distributions and model specifications, both in terms of bias and *RMSE*. This indicates that the inverse Wishart distribution may be a viable alternative under these conditions, and contradicts the frequent discouragement about its use in the literature (e.g., Gelman, 2006). The criticism is because in case of the inverse Wishart distribution, there are a-priori dependencies between the entries of the covariance matrix, i.e., between the variance components, and the variance components and correlations (Alvarez et al., 2016; Liu et al., 2016). Thus, the inverse Wishart distribution is likely to be highly informative in its standard diffuse specification and may lead to biased parameter estimates in case of large correlations and small variance components, and small correlations in connection with large variance components (Liu et al., 2016). Moreover, as the marginal distribution for the variance components is inverse Gamma, the inverse Wishart performs worse when the true variance component is near zero (Alvarez et al., 2016). Both characteristics make it likely to introduce unintended information into the analysis, which may lead to biased model parameters (Liu et al., 2016; Tokuda et al., 2012). Even though the typical/extreme variance components and correlations considered in this study do not point directly to such problems, the OH2PL provides increased control of the actual amount of information that is introduced into the analysis, and therefore should be preferred.

In sum, based on our results, we recommend using either the half-Cauchy or the Exponential distribution as hyperprior for the variance components, although the inverse Gamma distribution remains a viable prior choice if we know that the true variance component is sufficiently large. Their robust performance across specifications further allows for an increased flexibility and control when specifying prior distributions for variance components in hierarchical models. Since the differences in performance criteria are largely negligible, researchers will be able to focus on other criteria for choosing a specification. For example, we know that especially highly non-informative specifications are prone to convergence issues and sampling inefficiencies. We could show that there is nothing against using a more weakly informative specification of the half-Cauchy or Exponential distribution that aids convergence and sampling efficiency.

This is corroborated by the performance of the OH2PL regarding the bias in the estimated item and person parameters. Estimates of the item discrimination and the item difficulty estimates remained relatively unbiased across all simulation conditions, with clear advantages compared to the nonhierarchical 2PL and MML estimation in terms of *RMSE*. Although there was a slight increase in bias in the case of extreme variances, the average bias remained relatively small. This increase may be due to the underestimation of the respective variance components, as shrinkage towards the item parameter grand means should increase only when the variance in the individual parameters either decrease or is underestimated. This results in bias, because the true variance is actually larger, and the individual parameter estimates should not shrink towards their grand mean. It seems, however, that the underestimation of the variance components was small enough to cause no distinct bias in the individual parameter estimates. Thus, this suggests some flexibility with respect to the required accuracy of the elements of the variance-covariance matrix to obtain unbiased item parameters, and sheds new light on the theoretical relation between the variance components and the item parameter estimates in Bayesian hierarchical models.

The fact that the OH2PL outperformed its nonhierarchical counterpart provides a further indication that hierarchical Bayesian IRT models behave differently than hierarchical Bayesian models in general (cf. Koenig et al., 2020). From a theoretical point of view, bias in individual parameter estimates should always be slightly larger in the hierarchical model, due to the variance-dependent shrinkage effect. In the OH2PL, this was not the case. Another aspect that suggests the possibility of different behavior is the fact that the average bias in the item parameter estimates did not increase with an increasing variance component (at least for small and typical variances, and for the bias in the item difficulty estimates). Thus, the assumption of the different behavior of hierarchical Bayesian IRT models warrants further investigation.

We have to consider three limitations of the current study that open up pathways for further research on this topic. First, the half-Cauchy distributions exhibits convergence issues especially in its non-informative specification. It is possible, however, to apply a transformation based on the hyperbolic tangent function where the variance component is sampled from a uniform distribution bounded by zero and π/2 (as illustrated in the Introduction; Stan Development Team, 2022). As we did not implement this transformation in our design, it might be useful to investigate whether and how this transformation affects the convergence rate of the half-Cauchy distribution. Second, our results imply that both the inverse Gamma and inverse Wishart distributions may still be viable choices for hyperpriors in case (1) the variance components in question are sufficiently large and (2) the correlation falls into a certain range. Our simulation design, however, does not allow determining the critical values of the variance components and correlation. Here, we would need a more fine-grained resolution of the design, especially with respect to the true values of the variance components of the item discrimination parameters and the correlation between the item parameters. We will address this question in a future study. Third, in a similar vein, the current simulation design does not allow answering the question of the differential behavior of Bayesian hierarchical IRT models conclusively. This requires an extended and more fine-grained resolution of the design. We will address this question in a future study as well.

Summarizing, we showed that the performance of the OH2PL in its half-Cauchy or Exponential configuration is largely independent of the specification of the hyperprior distributions. This further strengthens the claim that the hierarchical Bayesian approach renders the 2PL IRT model applicable to small-sample situations that are typical in psychological research. In conditions with relatively few items and fewer than 100 respondents, where the estimation of item parameter variance is typically problematic because of sparse data, the OH2PL yields unbiased item and person parameter estimates. Moreover, as described in the Data Generation and Analysis section and further illustrated in our empirical example, the OH2PL shows fewer issues with convergence and inadmissible solutions compared to MML estimation. It does not need adjustments in the case of perfect response patterns. This shows that we can apply this state-of-the-art technique confidently across a broad range of empirical studies in which tests are used and that it presents resource-efficient possibilities for accurate item calibration under suboptimal assessment conditions.

## Data Availability

The datasets generated during and/or analyzed during the current study are available in the Open Science Framework (OSF) repository, 10.17605/osf.io/m3zaq.
